# Communication Network Architectures for Driver Assistance Systems

**DOI:** 10.3390/s21206867

**Published:** 2021-10-16

**Authors:** Romeo Giuliano, Franco Mazzenga, Eros Innocenti, Francesca Fallucchi, Ibrahim Habib

**Affiliations:** 1 Department of Engineering Science, Guglielmo Marconi University, Via Plinio 44, 00198 Rome, Italy; eros@newtechweb.it (E.I.); f.fallucchi@unimarconi.it (F.F.); 2 Department of Enterprise Engineering “Mario Lucertini”, University of Rome Tor Vergata, Via del Politecnico 1, 00133 Rome, Italy; mazzenga@ing.uniroma2.it; 3 Department of Computer Engineering, City University of New York, 160 Convent Avenue, New York, NY 10031, USA; habib@ccny.cuny.edu

**Keywords:** pedestrian detection, communication system, system information block, convolutional neural networks

## Abstract

Autonomous Driver Assistance Systems (ADAS) are of increasing importance to warn vehicle drivers of potential dangerous situations. In this paper, we propose one system to warn drivers of the presence of pedestrians crossing the road. The considered ADAS adopts a CNN-based pedestrian detector (PD) using the images captured from a local camera and to generate alarms. Warning messages are then forwarded to vehicle drivers approaching the crossroad by means of a communication infrastructure using public radio networks and/or local area wireless technologies. Three possible communication architectures for ADAS are presented and analyzed in this paper. One format for the alert message is also presented. Performance of the PDs are analyzed in terms of accuracy, precision, and recall. Results show that the accuracy of the PD varies from 70% to 100% depending on the resolution of the videos. The effectiveness of each of the considered communication solutions for ADAS is evaluated in terms of the time required to forward the alert message to drivers. The overall latency including the PD processing and the alert communication time is then used to define the vehicle braking curve, which is required to avoid collision with the pedestrian at the crossroad.

## 1. Introduction

The increase of traffic congestion in large cities is rapidly becoming a serious problem. According to [1], 12% to 23% of congestions are due to incidents along the roads. Moreover, in the near future self-driving vehicles will start to circulate on the road. Thus, both human drivers and autonomous vehicles will coexist and should be continuously aware of the local traffic situation to drive safe, to optimize the traffic flow and to avoid accidents with other vehicles and with pedestrians. The adoption of Autonomous Driver Assistance System (ADAS) can be helpful to support (human/autonomous) drivers by alerting them of possible imminent dangerous situations. Theoretically, the ADAS should be capable of i. acquire data in real-time concerning the area where the vehicles are moving, ii. use these data to analyze the current status of the area, and iii. predict the evolution of the status of the area so as to establish the potential occurrence of anomalous and/or dangerous situations. This analysis should account for the available information as well as on data collected from past experiences.

Recent works on the application of deep convolutional neural networks (CNNs) showed that CNNs are very promising tools for automated object detection in videos and images, especially for face recognition as well as for pedestrian detection (see [2] and references within). Current assistance systems recur to the use of CNNs in the detection of deformable objects such as pedestrians. Most of them acquire images by car-mounted sensors or video cameras, that are capable of detecting crashed cars or pedestrians and other objects on the road [3]. These systems can also predict the position of the objects on a short term basis. Thus, they can support the driver in limited visibility situations (e.g., night, fog), but since the camera is typically mounted on the car roof, the scene captured by sensors is the same of the driver. This strongly reduces the effectiveness of the assistance system especially when the pedestrian is moving behind a street corner. Other assistance systems are based on sensors, such as surveillance cameras, installed along the road (e.g. on street lamps) for monitoring the traffic flow, detecting vehicle incidents, and/or assessing the violation of traffic rules [4]. In all previous cases, the assistance system is only helpful for providing support to crashed vehicles or for sanctioning traffic violations, but it is ineffective for preventing incidents involving drivers and pedestrians.

In this paper, we propose a driving assistance system to warn human drivers and/or autonomous-driving vehicles on the presence of pedestrian crossing the road in those areas where the visibility at the crossroad is reduced. The basic system requires the installation of one video camera in the proximity of the selected crossroad so as to have full visibility of pedestrians moving in the crossroad area. The adoption of more cameras can be considered when the crossroad requires a more detailed image acquisition. The camera is then connected to a pedestrian detector (PD) sub-system whose main functions are to acquire images from the local area and analyze them to detect the presence of pedestrians. To this purpose, the PD should perform the following tasks: distinguishing objects from the background; recognizing if a candidate object is a pedestrian or not; localizing the detected pedestrians. Several pedestrian recognition systems were proposed in literature (see for example [5,6]). The PD presented in this paper is based on the YOLO CNN [7] which is suitable for this application. Since fast processing speed is a stringent requirement, other methods, such as Histogram of Oriented Gradients and Support Vector Machine, were discarded since they are slow and the detection accuracy is poor when the image resolution is low [5].

When crossing pedestrians are detected and localized, the PD generates the alert message and sends the collected information to the intended vehicles approaching the surveilled area. Alert messages and other information can be transferred to vehicles using one of the three alternative communication infrastructures, which are detailed and analyzed in the following of the paper. In the first and second solutions, we consider the possibility of reusing existing 4G (or on the forthcoming 5G) mobile network or fixed access networks to connect the PD and the remote server running the driver assistance application. In the first solution, the driver receives alerting messages by the remote server using the standard emergency warning system (EWS) [8] supported/implemented by the telecommunication network operator (Telco). Instead, in the second solution, we assume the remote server acts as a message broker. In this case, the received alert message from the PD is relayed to the vehicles that subscribed to the assistance service and are connected to the broker by means of the public mobile networks. Finally, in the third case, we consider a distributed communication infrastructure. We assume each PD equipped with a corresponding camera can send the alert message directly to the incoming drivers by means of Wi-Fi/IEEE 802.11p or any other local area radio technology. As shown in the following of the paper, this solution allows to drastically reduce the latency required for the forwarding of the alert message to the drivers. The main aim of this work is to propose three types of telecommunication systems based on who manages the information providing the ADAS service (i.e., the Telco, an external service provider/broker, usually called Over-The-Top, or a local manager) and where the information itself is processed (i.e., in a remote server or in a local edge computing server). Furthermore, we analyze the most suitable telecommunication architectures in terms of deployed elements and latencies to provide information to the driver (i.e., the crossing pedestrians detected by the PD) so as to guarantee the required delay. The proposed analysis is to be considered independent of the type of neural network adopted by the PD, indeed it can be replaced at any time with the latest updates in the field.

The paper is organized as follows. In Section 2, the main related works on driver assistance systems and pedestrian detection are reviewed. In Section 3, the considered scenario of a typical crossroad and the principle scheme of the proposed assistance system are detailed. In Section 4, the communication infrastructures supporting the considered ADAS system are described. In Section 5, we analyze the performance of the considered communication architectures able to provide the driver with warning message of crossing pedestrian. In Section 6, the CNN-based PD is presented and the related testbed is discussed. In the same Section, metrics and performance results of the PD are reported. In Section 7, we define and discuss the braking curve accounting for the overall ADAS latency to be applied to vehicles approaching the crossroads. Finally, conclusions are given in Section 8.

## 2. Related Work

The problem of supporting drivers in detecting moving obstacles on the road was studied for several years in many different aspects, concerning traffic monitoring, car accidents, and crossing pedestrian detection [9,10]. Systems concerning traffic monitoring and the identification of the root causes of traffic, such as obstructions of not well parked vehicles over the road, are able to communicate with other cars and with the infrastructure to propagate this information to other vehicles so as to improve safety [11,12]. Several works were presented in the literature concerning the detection of crossing pedestrian. Authors in [13,14] produced significant performances addressing this topic. A comprehensive review of the predicting pedestrian behavior in urban scenarios research is presented in [15]. All these approaches for pedestrian action prediction exploit the standard pedestrian detection strategy, which only discriminates between pedestrians from nonpedestrians. In [16], authors propose a method for prediction of pedestrian crossing intention by comparing images at different timestamps.

Many others proposed solutions are usually based on car-mounted cameras to support the driver. In [17], authors propose a three-level, coarse-to-fine, video-based framework that detects partially visible pedestrians just as they enter the camera view. The system provides low false alarm rate and operates very fast in pedestrian recognition. The work in [3] focuses on sudden pedestrian crossing at night using images gathered by a far-infrared camera. Other works as in [18] proposed a method for predicting the crossing intention of a pedestrian on a sidewalk based on the pedestrian’s posture and change in posture in multiple image frames during a short observation time period. In [19], authors provide a dataset for the classification of pedestrian movements and their intentions according to their motion direction using deep learning techniques (http://www.rovit.ua.es/dataset/pedirecog/, accessed on 15 October 2021). New recent research is related to detect pedestrians on high-speed roads using high-resolution images [20] showing that ADAS is of great importance for preventing collisions especially when autonomous vehicles are considered.

Papers in the literature mainly focus on the pedestrian detection functionality striving to continuously improve its performance under variable and difficult operating conditions. Instead, in this paper we define the architecture of an ADAS able to provide a pedestrian alerting service to vehicles approaching crossroads. The pedestrian detection feature is only one component of the considered ADAS, which also includes all the communication facilities and functionalities required to forward alert messages to the drivers approaching the crossroad. The main innovative aspects in the proposed ADAS can be summarized in the following points:

- test of a specific CNN-based solution for the practical implementation of the pedestrian detection subsystem, including the design of the related setting parameters to guarantee the required timeliness of the warning message delivery;
- definition of three different communication network architectures able to deliver the alert messages generated by the PD to the intended vehicles; among them, an entirely distributed communication infrastructure using local area wireless technologies was presented and analyzed in this paper;
- discussion on efficient solutions for managing and delivering alert messages to the intended users in the crossroad area for each one of the considered communication infrastructures for ADAS; to this purpose the concepts of local relevant area and of extended crossroad area were introduced;
- a format for the alert message to be broadcast using the emergency warning system embedded into the public mobile networks was presented.

## 3. Driving Assistance System

### 3.1. Operational Scenario

**Figure 1:**
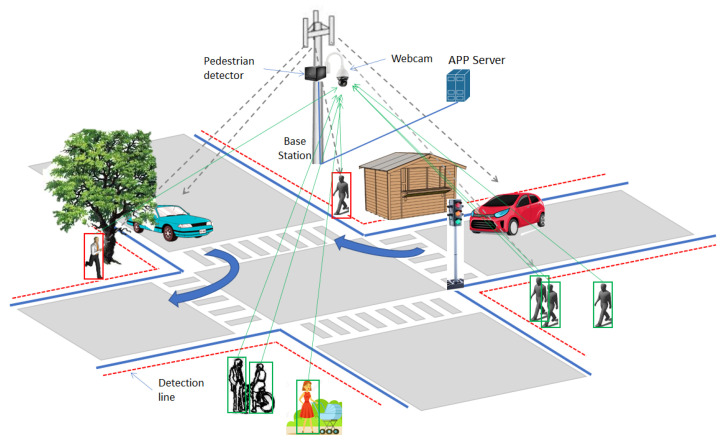
Typical crossroad scenario.

The typical scenario adopting the proposed driving assistance system is depicted in Figure 1. The system includes the camera in Figure 1, monitoring the crossing area. The video/image is then provided to a PD subsystem, which processes these data to identify pedestrians crossing or willing to cross the street. In the case of crossing detection, the PD generates and sends an alert message to one (remote) application server (AS). The message can include the actual spatial coordinates of the detected pedestrians crossing the street.

The AS forwards the alert message to the apps running on the terminals inside the vehicles approaching the crossing area. The apps use these information to support the driver’s activity as well as the self-autonomous driving system (if any).

As a general rule, the PD classifies moving persons in the crossing area into two categories: i. those leaving or that are going to leave the sidewalk (indicated with a red bounding box in Figure 1); ii. persons on the sidewalk not crossing the street (indicated with green bounding box in Figure 1). The green bounding box of each pedestrian leaving the sidewalk and crossing the dashed (virtual) line in Figure 1 changes into red. The bounding box remains red until pedestrian exits the crossing zone i.e., alert messages are sent to the AS until the surveilled crossing zone returns to be clear. In this last case, one message to clear the alert condition can be sent from the AS to the intended drivers.

Vehicles approaching the crossing area and receiving an alert message become aware that someone is occupying the lane. Additional information of the coordinates of the crossing pedestrians could be used to identify even the occupied lane in the crossroad. This information could be used to inform drivers of the presence of persons on its lane in the case of obstructed view. As an example, referring to the case in Figure 1, due to the presence of the tree, the driver in the (turning) blue vehicle cannot see the person crossing the street.

### 3.2. ADAS Principle Architecture

**Figure 2:**
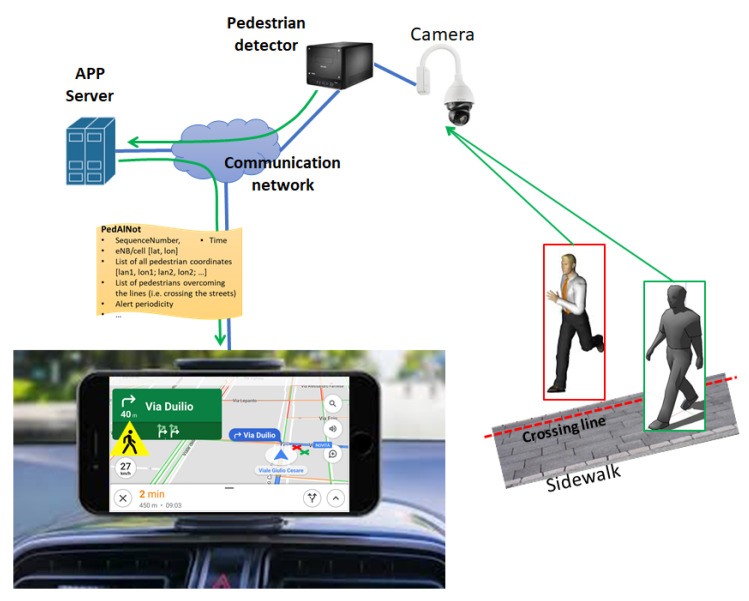
Principle scheme of driving assistance system.

Figure 2 reports the principle scheme of the system architecture for pedestrian detection and warning service. As shown in Figure 2, the building blocks of the considered driving assistance system are listed in the following points:

- the video camera monitoring the crossing area (installed in a favorable position);
- the PD, which classifies pedestrians and may possibly generate alerts in the case of crossing;
- the communication infrastructure required for the PD to communicate with the AS and for the AS to alert vehicles;
- the app running on the smartphone/tablet of the driver or running on the device(s) for the autonomous driving.

In the architecture in Figure 2, it was implicitly assumed the processing in the PD is executed locally by one subsystem directly connected to the camera. In principle, part or the processing in the PD could be executed in the APP server residing in the cloud or at the edge (i.e., the MEC). This allows to drastically reduce the hardware/software complexity of the camera with the PD subsystem to be installed at the crossroad. This is achieved at the expense of an increased transmission bandwidth (depending on the image resolution) required for the camera(s) to send images to the remote server.

Considering the scheme in Figure 2, in the following Section we discuss some viable architectures of the communication infrastructure allowing PDs to send warning messages to the intended recipients.

## 4. Analysis of Solutions for the Communication Subsystem

For the ADAS to be useful, the communication infrastructure used to inform vehicles approaching the crossing area is of importance. In this Section we detail and analyze three different solutions for the ADAS’s communication subsystem. The first two are based on the usage of existing 4G/5G mobile radio networks. The third one is based on a distributed approach where alerts are directly transferred to vehicles using a local area communication technology such as WiFi, direct WiFi, or IEEE 802.11p. In this case, 4G/5G networks could be used for remote control/setting of the PDs and orientation of cameras. In the first two cases, data collected by cameras are processed by the PDs. Alert messages generated from PD are then forwarded to the AS and then to the intended vehicles using all the available public mobile radio networks. The AS is in charge to (eventually) manage the received alerts with pedestrian data and to intelligently redistribute them to the vehicles approaching the crossing area.

### 4.1. Communication Subsystem Based on Existing Warning Systems in the Mobile Networks

**Figure 3:**
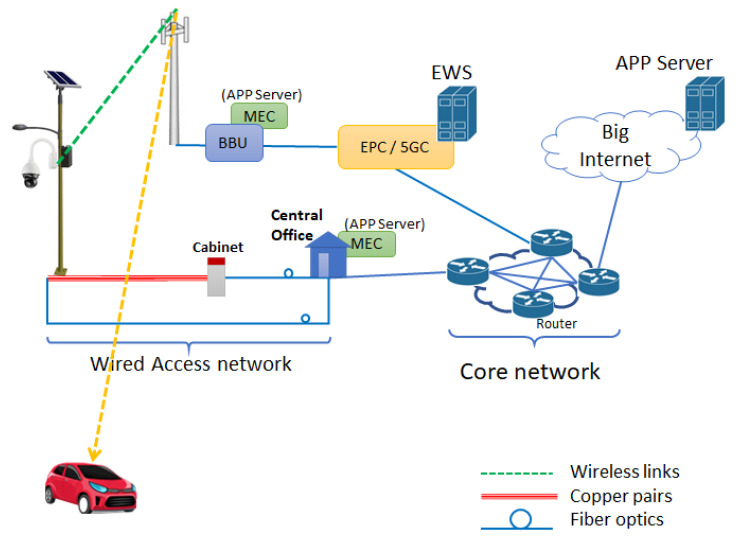
Architecture of communication system based on existing 4G/5G mobile and fixed access network with EWS.

As shown in Figure 3, the PD transfers alert messages possibly including additional data using the radio link provided by the eNB (or gNB) covering the area. As an alternative, the PD could be connected to the rest of the network using a copper pair or a fiber optic. Alert messages are received by the remote AS that retransmits them to the mobile warning system, with the indication of the coordinates of the PD generating the alert.

The message received by the warning system is routed to the eNBs/gNBs covering the crossing area in which the PD is located. Finally, the eNB/gNB can use the broadcast channel (BCH) to provide alerts (and other pedestrian information) to drivers entering the surveilled area. Typical information sent through the BCH by the local BS are information for Public Land Mobile Network (PLMN) selection, cell selection, and intrafrequency cell reselection. In the LTE case, the System Frame Number, the Channel Bandwidth, and Physical channel Hybrid/ARQ Indicator Channel (PHICH) information are included in MIB (Master Information Block). Instead, data for supporting the intrafrequency reselection are included in SIB (System Information Block) type 1 to type 4. Interfrequency cell selection or cell reselection with other cellular systems (i.e. UTRAN and GERAN) are sent in SIB type 5 to type 7. Another example of message broadcast is the Wireless Emergency Alert (WEA), formerly known as Commercial Message Alerting Service (CMAS) in US, which uses SIB-12 for broadcasting messages. WEA/CMAS is a public warning service developed for the delivery of warning notifications during emergency situations (e.g. earthquake, Tsunami, Presidential, and child abduction emergency alerts). This service uses the Cell Broadcast Center to send emergency notifications to users located in the selected cells. Based on this feature of modern mobile radio technologies, we propose to define a Pedestrian Alert Notification (PedAlNot), to be transmitted on one of the SIB messages (e.g., SIB-14 or higher). PedAlNot provides on-board devices with the information on pedestrians’ position. The following information can be included in this message:

- Message ID: it identifies the (specific) crossroad;
- Enable/disable: it specifies if the PedAlNot is active (and the info in this message can be considered as true);
- Sequence Number: identifies variations of a PedAlNot. This number increases for each new message, so vehicles can discard the older ones;
- Time: reports the sending timestamp of the alert. Vehicles can discard old notifications, even if they are sequentially valid;
- eNB/cell antenna position coordinates: it contains the [lat, lon] coordinates of the sending cell antenna. It helps the receiver to correctly display the info;
- Number of affected areas: it provides the number of affected areas, which are included in this message. Each of them provides information on pedestrians located in that area;
- Affected area #1: it consists in a circular area, defined by the [lat, lon] coordinates of the center and its radius. The on-board device can discard it whether its GPS position is outside this area;
- Alerting pedestrians position coordinates of affected area #1: it reports the [lat, lon] coordinates of all the pedestrians, who have crossed the line and therefore are going to cross the road. Nearby pedestrians can be grouped together reporting the same position, to simplify notification;
- …
- Affected area #N: center and radius of this area;
- Alerting pedestrians position coordinates of affected area #N;
- Alert periodicity: it reports the updating time the alert is sent. It allows to know the refresh time info.

In Figure 2, the on-board smartphone screen displays the two detected pedestrians represented by one green and one red “X”. The big yellow sign on the screen indicates the (generic) presence of a crossing pedestrian.

**Figure 4:**
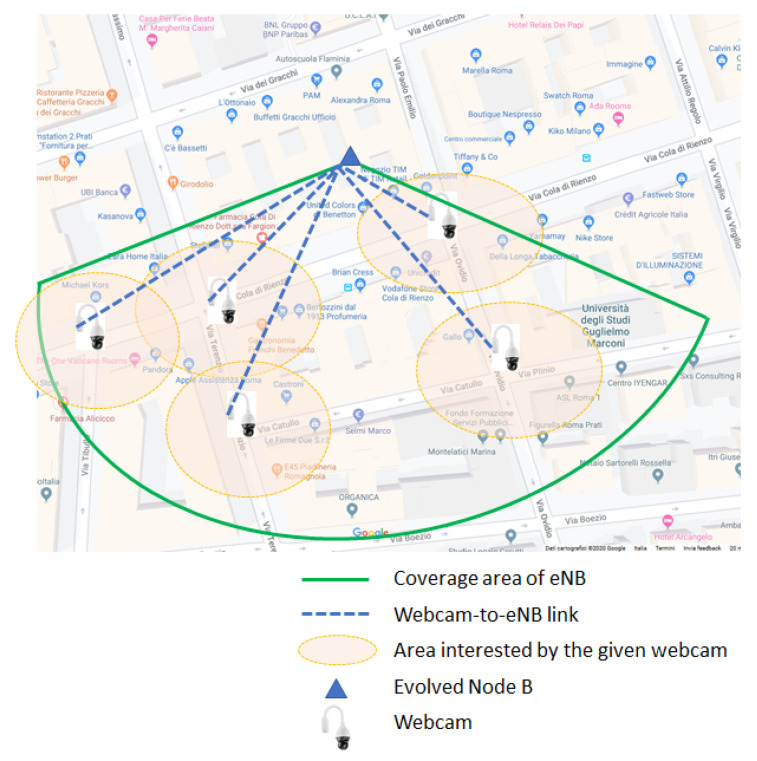
Affected crossroad areas served by same eNB.

In the example in Figure 4, we report the case of one eNB covering one area containing five crossroads. For each crossroad, it is possible to define a *local relevant area* (LRA), represented by a small circle (radius of tens of meters) over the crossroad, in which alert information are assumed to be valid. The on-board vehicle device receives all the PedAlNot messages. For each alert message, the on-board app compares the crossroad center/radius with the position of the vehicle to determine if the considered message should be discarded or not. If the message is considered to be relevant for that device, the pedestrian positions are visualized and the driver alerted even with an acoustic signal.

### 4.2. Communication Subsystem with a Message Broker Server

The communication system architecture is identical to that in Figure 3. The PD with camera is connected to the AS by using the existing public mobile radio network(s) or the copper/fiber-based fixed access network(s). The AS processes the received messages and, differently from the previous case, it operates as a message broker server able to provide notifications to apps. In the following, we indicate this server as AS broker. In this case, the main functionalities of the app running on the vehicle’s device are summarized in the following points.

- The app registers with the AS broker for subscription to the ADAS service;
- Data connection between the app and the AS broker is maintained active while the vehicle is moving. To this purpose the app periodically sends one registration update message to the AS broker. This message could include the current position, the vehicle speed as obtained by the on-board GNSS receiver.

When the AS broker receives the alert message from one camera, it forwards the alert to all vehicles moving in the *extended area* defined around the camera. On the basis of the exact position of the vehicle, the corresponding app decides to keep or discard the alert message notified by the broker. In particular, if the app is aware the vehicle is approaching the crossroad area indicated in the alert message, the notification is shown to the driver, otherwise it is discarded. The app running in the on-board device also receives the message to clear the alert condition when the crossing area turns out to be free. The possibility for the AS to act as a message broker allows the proposed ADAS to operate independently of the availability of the emergency system of the mobile operator network as in Figure 3.

**Figure 5:**
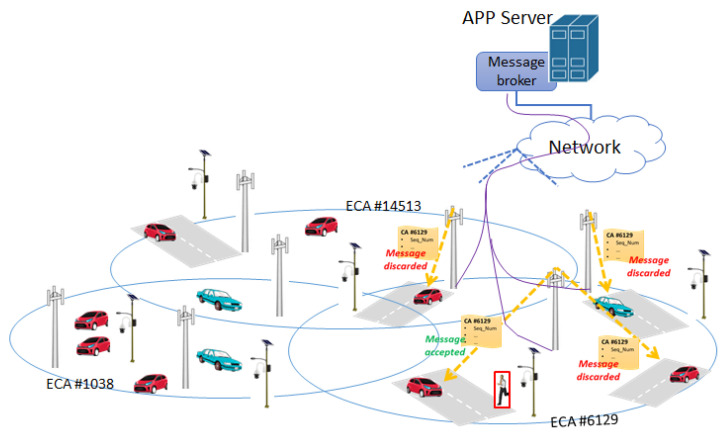
Scheme of system architecture in presence of a broker properly forwarding PedAlNot messages.

To avoid the AS broker broadcasts alerts to all the vehicles in the entire ADAS service area, we propose that the alerts from one camera are sent only to vehicles traveling in the extended crossroad area (ECA), which is specific to each camera. The vehicles in the ECA of one camera are identified on the basis of their positions and possibly vehicles’ speeds, which are provided to the AS broker by the periodical keep-alive messages from vehicles used to maintain the data connection active. Taking into account this information on the time elapsed from the reception of the last update message from each vehicle and of the street topology, the AS broker should be able to identify vehicles moving into the ECA associated to the camera that could be interested in receiving the alert message. Since not all vehicles in the ECA are directed to the crossroad surveilled by the alerting camera, some of them will discard the received alert message. To better explain this concept of ECA and its utilization in the ADAS we refer to Figure 5.

The AS broker forwards the alert message to all vehicles in the ECA associated to the alerting camera. As an example, looking at Figure 5 the alert from the camera in the ECA #6129 is sent by the AS broker to all vehicles in the ECA. In particular, the alert message is transmitted by the BSs covering the ECA using the data connections of the registered vehicles. The app in the vehicle, which is not moving toward the alerting camera, discards the received alert on the basis of its actual position and direction. In Figure 5, we highlighted in green the acceptance of the alert message by the app running in the vehicle directed toward the alerting camera; conversely, the vehicles indicated by red discard the alert message. The ID of the ECA can be assigned/generated by the AS broker. The extension of the ECA can be estimated on the basis of the map of the crossing areas. The list of vehicles in each ECA is updated every time the AS broker receives the data connection update messages from the connected apps. The considered ECA-based approach prevents the AS broker to broadcast the alerts to all vehicles in the ADAS service area. Furthermore, it can be observed that by adopting the considered strategy, it is not necessary for the AS broker to know the exact position, direction, or the identity of the vehicles specifically moving toward the alerting camera. The adoption of the ECA-based approach for the smart forwarding of the alert messages allows to improve ADAS scalability over large areas. In this second communication solution, the format of the alert message is up to the ADAS designer. The PedAlNot message format detailed in the previous Section could be adopted or used as a basis for defining another message format possibly accounting for some other specific service requirements.

As indicated in Figure 3, the usage of the Multi-Access Edge Computing (MEC) facility could be helpful to: i. reduce the broadcasting/multicasting latency of the alert message and ii. to perform CNN computation. This allows to exclude the pedestrian detection functionality in the device to be installed at the crossroad thus saving implementation costs.

### 4.3. Distributed Communication Subsystem

**Figure 6:**
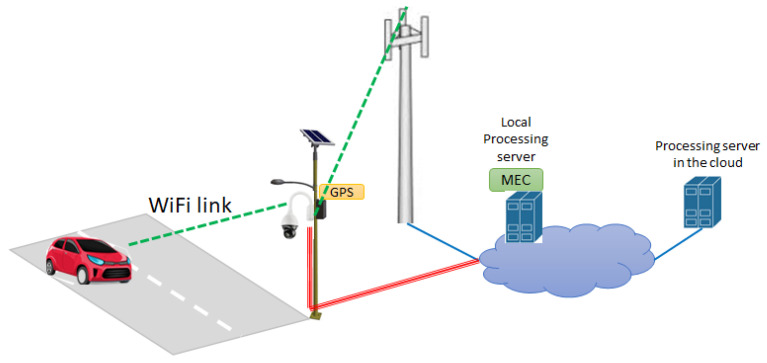
Principle scheme of distributed communication architecture.

An alternative distributed architecture for the communication system was depicted in Figure 6.

The single PD with camera generates the alerts, which are broadcast to the apps in the vehicles using a local radio transmission technology such as WiFi (or the IEEE 802.11p for equipped vehicles). For this arrangement to properly work, it is necessary the close interaction between the PD and the vehicle’s app as indicated in Figure 6. In particular, the main functionalities to be implemented in the PD and in the vehicle’s app are detailed in the following points.

- Positions and the working status of the PDs in the ADAS service area are periodically reported and updated into an electronic map. In the PD setup phase as well as during the normal operations, the single PD notifies its position (obtained from the GNSS) to one remote server. The main purpose of this server is to update the electronic map with the positions of the cameras and to store it.
- The (updated) map is downloaded by the apps in the vehicles. Map updates can be notified in real-time by the remote server, even acting as a message broker, to all the ADAS registered apps using the mobile radio networks in the service area. As an alternative, the app periodically checks for the map updates. This last approach simplifies the entire communication system and avoids the remote server to act as a broker. Nevertheless, in this case, it is not possible to notify the update of the map (if and when necessary) in real-time. This can be limiting especially when some cameras can go out of order.
- Apps into vehicles, approaching the crossroad area monitored by a given camera, start searching for the WiFi/ 802.11p radio link used by that camera itself for transmitting the alert message; to speed up this “synchronization phase”, information on the radio channel used by the camera could be stored in the map.
- The selection of the (next) camera to connect to is carried out by the on-board app using position, speed, direction etc. of the vehicle and the downloaded map of the area.
- The available public mobile radio networks could be used to exchange the corresponding management/control messages to check the status of cameras in the ADAS service area in real-time. In this case, it is assumed cameras are equipped with the corresponding mobile radio access terminals. Public mobile radio networks could also be used for the downloading of the (updated) map in the vehicles.

In the distributed communications subsystem in Figure 6, the latency for the local broadcasting/multicasting of the alert is drastically reduced with respect to the network solution in Figure 3 and, in addition, the proposed solution allows to bypass Telco’s network for the transmission of alert messages.

To reduce the implementation costs of the PD in the cases in Figure 3 and Figure 6, the CNN software could run on a remote processing server in the cloud, thus achieving PD computational offloading. As an alternative, it could run on a local MEC server. This allows to significantly reduce the communication latency, which is important in critical applications such as the one considered in this paper.

## 5. Latencies Performance for the Considered Communication Architectures

The overall latency of the considered ADAS is the sum of the following two contributions:

- *processing time*$\tau_{P}$ i.e., the time required to process the video/images from camera including the (possible) generation of the alarm message;
- *communication time* of the alarm $\tau_{C}$ i.e., the sum of the time required for sending the images to the processing server and of the time for sending (any) alarm message to the intended vehicles within the considered communication network.

In this section, we compare the considered communication network architectures in terms of the communication time. The exact formulation of the communication time $\tau_{C}$ depends on the position of the processing server in the network and the time required to return the alarm message to the intended drivers.

**Figure 7:**
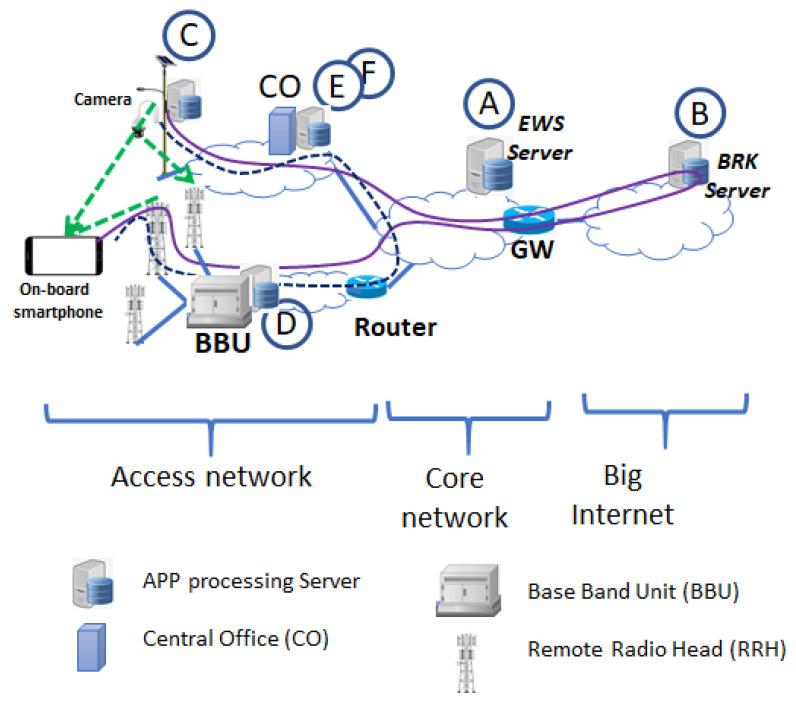
Network scheme for performance evaluation purpose.

To evaluate $\tau_{C}$, we consider the communication paths highlighted in the principle scheme in Figure 7. The communication paths differ according to the location of the processing server in the network. Each path may traverse different sections of the communication network i.e., the access network (including fixed and wireless links), the core network and the internet, which is outside of the Telco networks.

From Figure 7, we can evidence six main communication paths, labeled from *A* to *F*. To avoid a confusing picture, in Figure 7 only Paths B (solid line) and F (dashed line) were reported as two important examples. Paths and the corresponding latencies are detailed/evaluated in the following list:

- Path A: it refers to the communication Architecture 1; images are processed to the EWS server located within the Telco network; the emergency control channels (e.g., SIB-12) is used to send the message on crossing pedestrians to the drivers. In this case the $\tau_{C}$ is:
  (1) $$\tau_{C}=T_{wiredACC}+2\cdot T_{core}+T_{routBBU}+T_{air}+T_{app}$$
  where $T_{wiredACC}$ is the transmission delay in the fixed access network between the camera and the central office (CO), $T_{core}$ is the delay through the core network (i.e., from CO to EWS server). $T_{routBBU}$ is the time needed to cross the wireless access section from the delimiting router to the Base Band Unit (BBU). $T_{air}$ is the time for transmission from the BBU to the on-board terminal. It accounts for signal processing functionalities at the BBU and inside the user terminal and it includes the scheduling time in the eNB as well as the frame alignment. We considered this time can vary between a minimum and a maximum depending on the communication access technologies. Finally, $T_{app}$ is the processing time for the on-board app to process the alarm message and to visualize information to the driver on the screen.
- Path B: it refers to the communication Architecture 2; images are processed in the BROKER server located in the Internet (i.e., outside the Telco network). In this case $\tau_{C}$ is:
  (2) $$\tau_{C}=T_{wiredACC}+2\cdot T_{core}+2\cdot T_{Internet}+T_{routBBU}+T_{air}+T_{app}$$
  The $\tau_{C}$ in (2) is similar to $\tau_{C}$ in (1) except for the additional time $T_{Internet}$ required to traverse (twice) the internet to reach the message broker server and to return at the entry point of the wireless access network. In this case, the time in the air interface $T_{air}$ is higher than in (1) (see also Table 1) because the alarm message is sent to the drivers using the user channels, i.e., in this case the emergency service provided by the Telco for sending the alarm is not considered.
- Path C: it refers to the communication Architecture 3; images are processed locally. In this case $\tau_{C}$ is:
  (3) $$\tau_{C}=T_{camWiFi}+T_{WiFiAPP}+T_{app}$$
  where $T_{camWiFi}$ is the time required for the connection between the camera and the Wi-Fi modem, and $T_{WiFiAPP}$ is the time to transmit the alarm to the intended driver through the Wi-Fi link.
- Path D: it refers to the communication Architecture 2 including MEC; images are processed inside the MEC located in the BBU. In this case, the camera is connected to the eNB to send images to the server in the MEC and $\tau_{C}$ is:
  (4) $$\tau_{C}=2\cdot T_{air}+T_{app}$$
  The alarm is sent to the intended driver using the eNB of the cellular system. Even in this case, we assume that eNB downlink data channel is used to send the alarm.
- Path E: it refers to a variation of the communication Architecture 3; images are processed in the MEC located in the CO (i.e., in the wired access network). In this case, $\tau_{C}$ is:
  (5) $$\tau_{C}=2\cdot T_{wiredACC}+T_{WiFiAPP}+T_{app}$$
  The $T_{wiredACC}$ is the time interval required to send back the alarm through the wired access network, before the Wi-Fi access point.
- Path F: it refers to Architecture 2; the processing server is located in the MEC inside the CO. Alarms are sent to the intended driver through the eNBs of the wireless access network. In this case, the $\tau_{C}$ is:
  (6) $$\tau_{C}=T_{wiredACC}+T_{core}+T_{routBBU}+T_{air}+T_{app}$$
  Even in this case, eNB downlink data channels are used to send the alarm.

In Table 1 we have detailed the minimum and maximum latencies, expected for each component of the considered communication path. Data were taken from [21,22]. Note that for Architecture 1 (path A) and Architecture 2 (path B, path D, path F) we have considered 4G-LTE in the air interface time (i.e., the worst case). Further latency reduction occurs when 5G is available as access technology, giving a $T_{air}$ of about 1 ms or even below when self-contained frames and short time transmission intervals are used (e.g., for 60 kHz and 120 kHz carrier spacing), instead of 3–18 ms typical of LTE Release 8.

**Figure 8:**
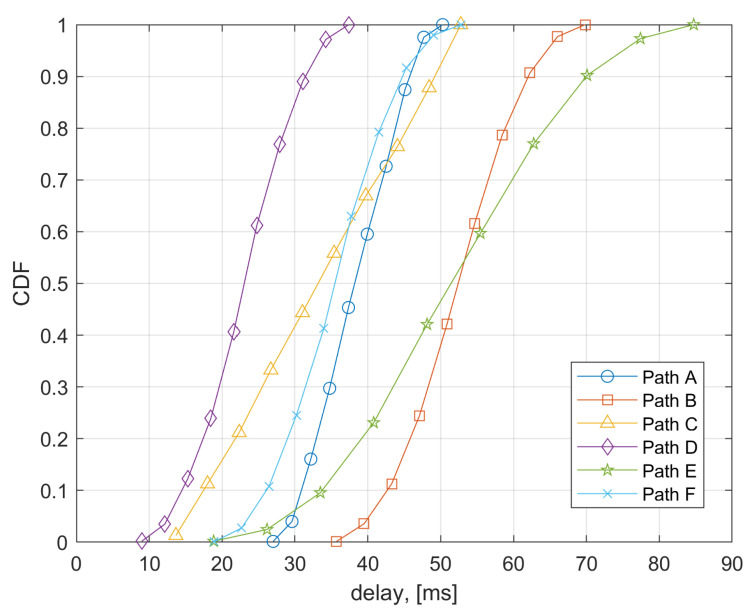
Cumulative Distribution Function of latencies in considered communication system architectures.

The Cumulative Distribution Function (CDF) of the overall delay on each path was considered to assess performance. This is the sum of the latencies for each link, which are assumed to be independent random variables uniformly distributed between the corresponding minimum and maximum values in Table 1. Results on CDFs are reported in Figure 8 for the three considered system architectures specified in Section 4. Moreover, we also reported the cases corresponding to the usage of MEC for processing the images.

From Figure 8 the performance of Architectures 1 in Figure 3 (EWS server, path A) and of Architecture 3 in Figure 6 (LOCAL, path C) outperform that of Architecture 2 in Figure 5 (usage of Broker server, path B) of about 20 ms on average. However, in any case all the considered communication architectures allow to obtain acceptable latencies for the ADAS service as also discussed in the next Section 7. The possibility of locating the image processing server in the MEC (EDGE cloud case, path D) allows to achieve a performance improvement in the communication latency. In fact, in the MEC case latency is mainly related to the access network segment. Performance improvement in the MEC case are of about 15 ms with respect to Architecture 1 and of about 30 ms with respect to Architecture 2. However, edge computing poses the challenges of energy and memory constraints. At this time, it is difficult to evaluate how the proposed PD processing deals with energy and memory problems in the MEC, since at present, the modalities of access and usage of the computational resources of the MEC are not well established, yet.

Considering as a reference overall delay the 90% percentile in each communication path, we observe the following overall delays $\tau_{C}$ for the given architectures:
      EWS = 47.1 msEDGE = 32.9 ms      BROKER = 63.8 msWiFi-EDGE = 73.3 ms      LOCAL = 51.2 msEDGE fxd/wirel ntw = 46.5 ms

In all cases, delays are always within 33 ms and 73 ms. Architectures in Figure 3 and Figure 5 exploit the already existing and future radio communication infrastructures (e.g., 3G/4G/5G etc.) and their associated services such as the emergency/ alerting service. This allows a very fast deployment of the proposed ADAS system and allows to adopt well-proven radio technologies available in the smartphones/tablets. Anyway, when MEC is included in the system, the overall latency of these alternatives is not too far from that of the solution in Figure 6.

Furthermore, local area radio technologies could be subjected to (intentional/unintentional) jamming/disturbs. To this purpose, methods for reducing interference in local radio access should be considered (i.e., automatic transmission channel selection/reselection). This is not the case for licensed mobile radio technologies (4G/5G), where spectrum status is monitored by Telco’s to detect the presence of unwanted interfering transmissions. Finally, when local area radio technologies become unavailable for some reason, alert messages generated by cameras in Figure 6 can be routed through the public mobile networks (i.e., path F). In this case, the solution in Figure 6 behaves similarly to those in Figure 3 and Figure 5.

## 6. Subsystem for Detection of Crossing Pedestrians

The objective of the pedestrian detection subsystem is the recognition of pedestrians crossing or willing to cross the street by means of the real-time analysis of a live video stream captured by the camera installed at the crossroad. Detection of pedestrians moving on streets is a primary and well-investigated task in many applications. During the last few years, deep convolutional neural networks (CNNs) were shown to be very promising for automated object detection from videos and images [23,24,25], as well as for the detection of deformable objects which is more akin to the pedestrian detection problem analyzed in this paper [2,26].

This section starts with the description of the operations of the considered CNN structure. Then, we detail its implementation and we describe the testbed used to evaluate performance of the proposed CNN-based PD.

### 6.1. Pedestrian Detector Subsystem

**Figure 9:**
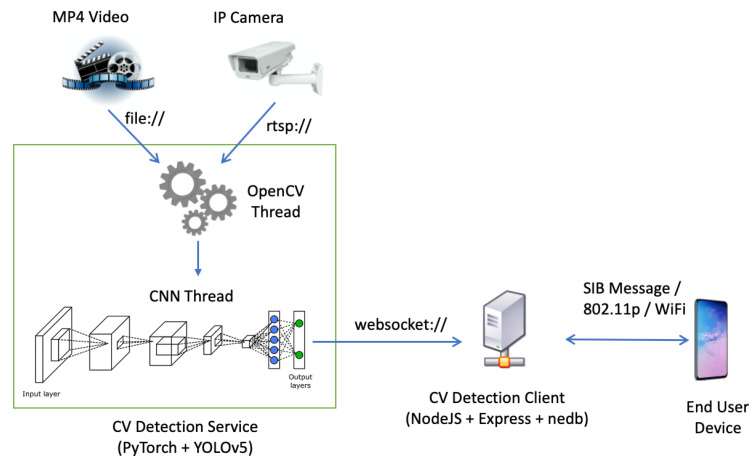
Scheme of PD implemented in testbed.

Effective machine learning approaches for object detection are based on multiple layer CNNs with the aim of extracting from an image its key features, such as edges or patterns, that can be subsequently used to classify objects that can be present in the image itself. In our setup, we selected the YOLO CNN [7] (further details are at https://doi.org/10.5281/zenodo.4154370, accessed on 15 October 2021) for the pedestrian detection application considered in this paper. As shown in Figure 9, the considered PD subsystem is composed by the cascade of the following components: the Computer Vision (CV) Detection Service (incorporating the YOLO CNN) and the CV Detection Client.

The CNN uses a pretrained model based on the COCO dataset [27], containing 80 object categories from over 330k annotated images. The YOLOv5 architecture is available in eight different configurations. Depending on the number of parameters and the video resolution as input to the neural network, it is possible to select the best configuration for the available computational power. Taking the requirements of this application into account, we selected YOLOv5, with an input resolution of 640×640 pixels or 1280×1280 pixels, to achieve a good trade-off between processing speed and accuracy.

After the CNN training phase, the pipeline describing the CNN operations is now detailed. Visual information from the camera is passed to the CNN for subsequent processing aiming at the detection of pedestrians. In more detail, the operations carried out by the CNN in the CV Detection Service element are reported in the following points.

1. Bounding box prediction: K-Means clustering algorithm [28] is used to find good priors instead of hand-picking them. The distance between centroids of found clusters is calculated using Intersection Over Union (IOU), which is independent of the size of the box. This approach leads to a lower error compared to the standard Euclidean Distance measure [29]. In addition, the latest versions of YOLO (i.e., v5 at the writing of this paper) use self-learning methods to derive most suitable anchor boxes for the input training data. By the means of logistic regression, YOLO predicts an objectness score to each bounding box.
2. Class prediction: every bounding box also predicts the classes that may contain. To achieve it, a multilabel classifier is used, whose main goal is to make prediction of the class type the object belongs to. YOLO predicts boxes using three different scales, ending up with a 3D tensor in the form of N×N×[3·(4+1+80)], where 4 are the bounding box offsets, 1 is the objectness prediction and 80 are the class predictions.
3. Feature extraction: YOLOv3 network uses successive 3×3 and 1×1 convolutional layers. Compared to that of the previous releases of the same network, this version is significantly larger and takes advantages from residual network technology. Residual networks offer the possibility to skip connections or jump over some layers improving the overall speed and accuracy. YOLOv5 further improves the feature extraction stage by using Cross Stage Partial (CSP) network as backbone [30]. CSP models are based on DenseNet [31], which are designed to address common CNN problems, such as vanishing gradient.

**Figure 10:**
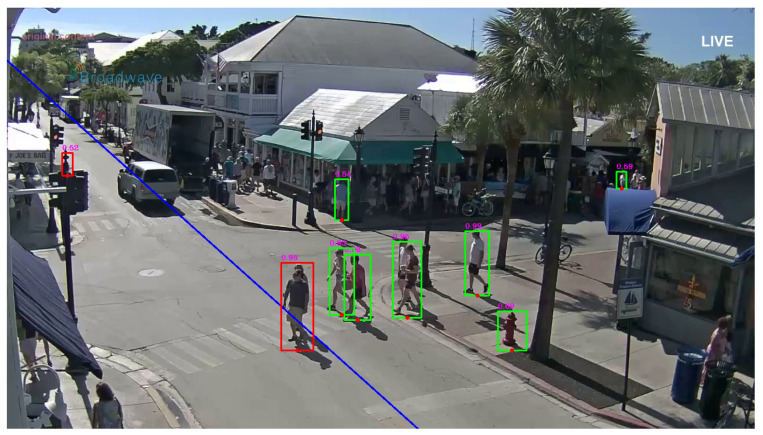
Frame example with bounded persons and set crossing line.

Finally, the CV Detection Client in Figure 9 separates into two groups the predicted pedestrian objects previously classified and “pictorially” surrounds them with a red bounding box if they are crossing the virtual line in the area and with a green box otherwise, i.e., the pedestrian is away from the crossing line indicated in the area (see Figure 10).

In the example in Figure 10, it is also reported the (blue) crossing line, which is set in accordance with the specific characteristics of the considered local crossing area. In Figure 10, it is possible to see the bounded persons recognized by the predictor with the corresponding indication of the assigned degree of certainty. The presence of green bounded persons are to the right of the crossing line and the presence of red bounded person just to the left of the crossing line.

### 6.2. Pedestrian Detector: Testbed Implementation

**Figure 11:**
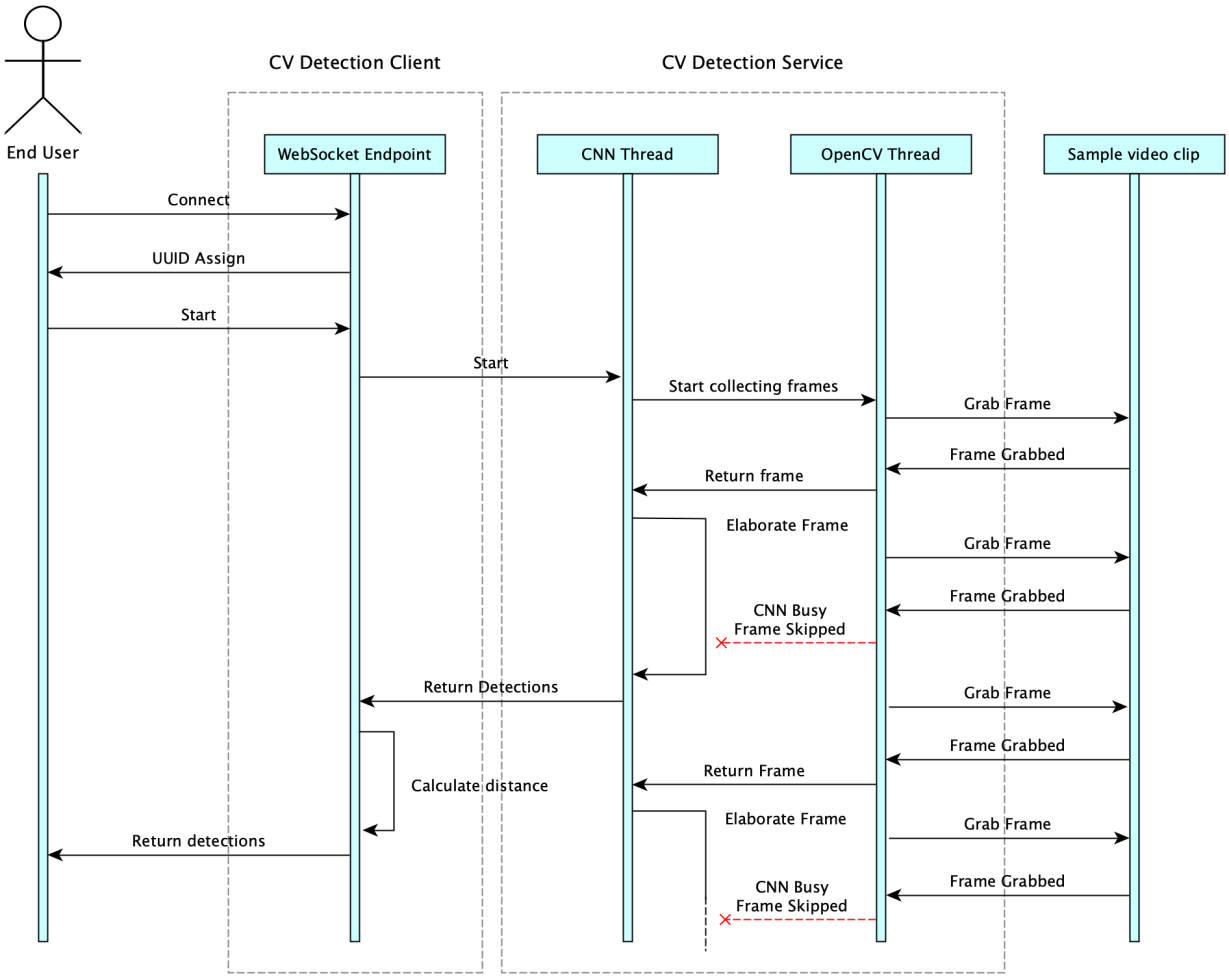
Activity diagram of PD for exchanged messages in warning service.

The CV Detection Service and CV Detection Client in Figure 9 may run on two separate systems as well as in the same host. The first one implementing the YOLO CNN is the most computationally intensive. Its operations can be represented by a multithread process in charge of grabbing and processing video frames captured by the local or remote camera. To guarantee strict real-time performance, this service should run on a performing workstation possibly equipped with a GPU (Graphical Processing Unit). Moreover, frames should be collected on a regular basis and the collection task should not be affected by the processing latency introduced by the CNN frame processing thread. This was obtained assuming that for each grabbed video frame, the service checks whether the CNN thread is running (thus, it is not available) or not (i.e., it is able to process a frame). In the latter, the new frame will be used as an input for the CNN. Otherwise, frame is skipped. The adoption of this implementation strategy for video processing allows us to keep the camera buffer always empty, thus making sure to prevent CNN to perform analysis on cached frames, which are no longer representative of the current situation in the local area. In Figure 11, it is reported the activity diagram for a single frame analysis, starting from the end user connection.

When the predictions are ready at the output of the CNN thread, a message is post to the CV Detection Client, whose task is to calculate the distance of the identified bounding boxes corresponding to pedestrians from the crossing line superimposed on the image of the local area as shown in Figure 10. Based on this calculated distance, the CV Detection Client decides whether the alarm should be issued or not. In the former, it notifies the application server (see Figure 3) of the potentially dangerous situation so to send the corresponding alert message to vehicles. As an alternative, in the case of Figure 6 the alert message can be directly sent by the camera to the local vehicles.

For the testbed implementation, we considered seven video clips (six of them publicly available on the web, while the seventh directly acquired in the field). Each video clip is characterized by different features such number of pedestrians, resolution, frame rate impacting/influencing the achievable accuracy, precision, etc., of the proposed PD as shown in the next Section.

### 6.3. Performance Metrics for Pedestrian Detector

Reactivity and reliability of the pedestrian detector are fundamental requirements for applications concerning people safety. A miss detection of a crossing pedestrian may lead to an accident. Reactivity of PD is also an important aspect because a fast warning system is a strongly desirable feature. In the following, we indicate the considered performance figures used to analyze the behavior of the proposed PD system. In particular, we evaluate performance of PD in terms of accuracy, precision, recall, and F-measure, which can be formally defined as:

(7) $$Accuracy=\frac{TruePositives+TrueNegatives}{TotalNumberOfObjectInTheFrame}$$

(8) $$Precision=\frac{TruePositives}{TruePositives+FalsePositives}$$

(9) $$Recall=\frac{TruePositives}{TruePositives+FalseNegatives}$$

(10) $$F_{Measure}=2\cdot {\left(\frac{1}{Precision}+\frac{1}{Recall}\right)}^{-1}$$

In our PD, the bounding box always (correctly or wrongly) indicates the position of an object classified as pedestrian. Four possibilities can be considered in the process of assigning the bounding box to an object. These are accounted for the performance figures in (7)–(10). True positives (TPs): is defined as the number of bounding boxes with pedestrians correctly classified by the CNN as pedestrians. False negatives (FNs) (i.e., miss detection of pedestrian): is the number of pedestrians that were not included in bounding boxes nor classified as pedestrians at all. False positives (FPs): the number of bounding boxes indicating nonpedestrian objects. Finally, true negatives (TNs): is the number of objects correctly classified as nonpedestrian. The total number of objects in the frame is the overall sum of the previous figures. The sum of TPs and FNs in (9) is the total number of pedestrians in the scene that should be identified and surrounded with bounding boxes. Thus, the recall is the number of correctly identified pedestrians in the scene divided by the number of all existing pedestrians in the same scene including those that were not identified by the CNN. Finally, note the TNs parameter is not important in the PD case, and it is always assumed as zero.

### 6.4. Performance Results for Pedestrian Detector

To analyze the performance of the proposed PD subsystem, the video stream provided by the camera was replaced by two groups of videos, representing some of the typical environments. The first group comprises simpler cases, such as less crowded scenarios and low to zero occlusions. The second group, conversely, refers to real-life videos, which were taken from public live cameras available on the internet or directly recorded in the field.

As previously outlined, clips are characterized by different video framing and camera angle/perspective. Four of them were taken from the Pedestrian Direction Recognition Dataset by Robotics & Tridimensional Vision Research Group (http://www.rovit.ua. es/dataset/pedirecog). These video clips were acquired using an outdoor stationary camera with a 640×480 pixel resolution. In these sequences, pedestrians are walking in different directions, sometimes overlapping each other.

In particular, the first and second video clips represent two simple scenarios in which people are walking in a single direction, from left to right (i.e., “Left”) and from right to left (i.e., “Right”) of the camera view. In “Multiple Directions” people enter the scene from both sides, thus overlapping each other more frequently as the previous video clips. “Zebra Crossing” frames a pedestrian crossing area where people are sometimes occluded by the cars passing by. The fifth clip (i.e., Florida) refers to a more complicated scenario. It was acquired by a public webcam placed in a crowded crossroad in Florida (US). Even though it is a high-resolution video, it is sometimes difficult to recognize pedestrians even by a human eye.

In “Av. Do Mar” we selected a 1${}^{\prime }$30${}^{\prime \prime }$ long video recording from a crossroad in Avenida Do Mar (Portugal). The last video clip was recorded by our work group in Rome, Italy. Videos from the real-life group differ from the simpler ones also for the resolution, which in these cases is equal to 1280×720 for “Florida” and FullHD 1920×1080 for both Av. Do Mar and Rome.

For each video, one sample of 50 consecutive frames was extracted. To evaluate the performance figures in (7)–(10), each sample was manually annotated with the ground truth bounding boxes. After running the PD on each sample, precision, recall, accuracy, and balanced F-Measure in (7)–(10) were evaluated, and results were reported in Table 2 for each clip taken into consideration.

During the benchmark execution, classification results with assigned degree of certainty below 0.5 was dropped, i.e., we assumed the classification was wrong and objects were not pedestrians. This alters some of the performance figures in (7)–(10). In general, results in Table 2 show that the simpler the scene, the better the metrics associated to the PD. Precision is close to 1 (or equal to 1) in all cases. However, when considering the main purpose of the proposed PD, the recall parameter is more important than precision. In fact, a missed detection is more important than a false positive. Note that in case the camera is properly deployed, recall presents high value close to 1, even if the video refers to a real-life environment (see Rome case). To compare results obtained by the proposed system with the typical ones available in the literature, we supposed to have the same system mounted on-board (e.g., the same camera and the same processing capabilities). In this case, the visibility of the scene is quite different and limited with respect to the case where the camera is well-deployed in a crossroad or close to zebra crossing area. Considering for example the Rome video, in case of on-board mounted camera recall and accuracy are 0.63, while precision and F-measure are 0.99 and 0.77, respectively. Furthermore, if we assume that the on-board system is equipped with a perfect object detector (i.e., zero FN and zero FP), recall and accuracy increase both to 0.65. Even in this case, the proposed system outperforms the other on-board detection systems (see the last row of Table 2).

**Figure 12:**
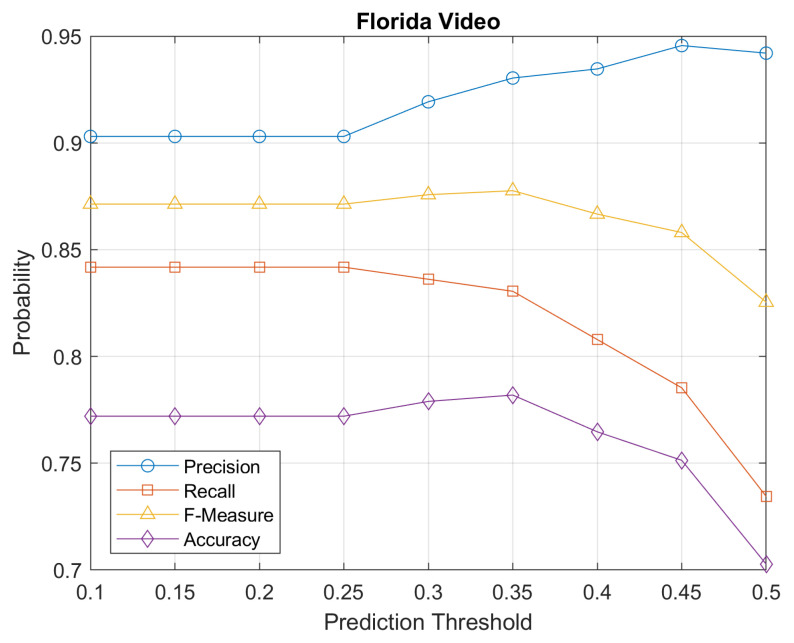
Metrics as a function of threshold of degree of certainty in Florida video.

The Florida clip presents false positives in some cases. One example is showed in Figure 10, where a fire hydrant is classified as pedestrian by the detector. This reduces the precision but this effect can be mitigated by properly selecting the frame dropping threshold based on the degree of certainty assigned by the classifier. This fact is analyzed in the following Figure 12, where we show the performance in in (7)–(10) for the Florida video clip, for variable certainty threshold from 0.1 to 0.5.

To examine the achievable real-time performance of the considered solution, we performed several benchmarks on the equipment available for testing. We started by considering the most complicated clip, called “Florida”, which is $1^{\prime }35^{\prime \prime }$ long and includes 2849 frames at 1280×720 pixels and a frame rate of 30 frame per second (fps). The Florida benchmark was conducted on a test machine with the following characteristics:

- Intel Xeon E3—1225, 4 core, 3.1 GHz processor
- 8 GByte RAM 1600 MHz DDR3
- GPU Nvidia GeForce RTX 3060 12 GByte RAM

All the available YOLOv5 configurations were considered in the benchmark. As mentioned in Section 6, this neural network architecture is provided in four different sizes (i.e., S, M, L, X). For each model size, two different input resolutions are available: 640×480 and 1280×1280, where the latter is also identified by the suffix “6”. It was proved that lighter models (i.e., S and M) have lower response times, thus allowing higher frame-rates at the cost of less accuracy. On the other hand, the input resolution parameter mainly affects the network accuracy on the basis of the object sizes, leading in better results when a high-input resolution is used for detecting small objects. In this case, given the position of the camera, we are interested in medium/large objects (i.e., pedestrians), therefore we picked YOLOv5 X, which has a lower input resolution. To assess real-time performance of the available arrangement, the experiment consisted in running the same input video on the same hardware and observing the following performance figures:

- Total Analyzed Frames (TAF), corresponding to the number of frames that have passed to the YOLO CNN;
- Total Unprocessed frames (TUF), corresponding to the number of non-analyzed frame or lost frames (i.e., it accounts for frames that were skipped because the YOLO CNN was busy); this is the difference between the total number of frames in the clip equal to 2849 in the considered video clip and the TAF;
- Total pedestrian Detections (TD), counting the bounding boxes returned by the CNN in the unit of time;
- Total Detections in Alarm zone (TDA), counting the number of bounding boxes in alarm state, i.e., red bounding boxes.

**Figure 13:**
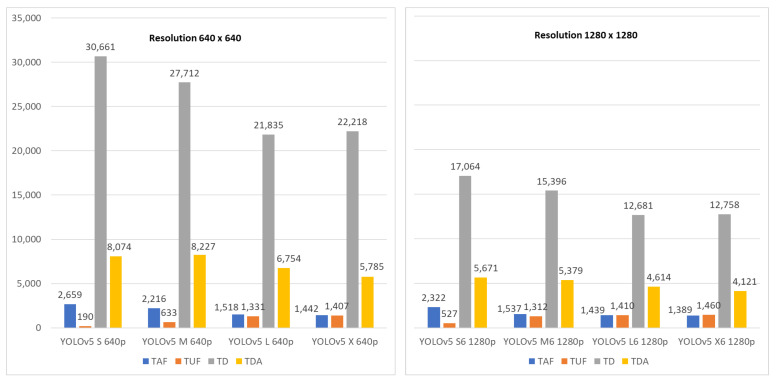
Analyzed frames and bounded pedestrians as a function of YOLO v5 CNN configuration.

Experiments were conducted considering variable fps depending on the image resolution and the model size. We obtained 26.8, 22.5, 15.6, and 14.7 fps for YOLO v5 S, M, L, and X respectively, while 23.6, 15.5, 14.5, and 14.0 fps for YOLO v5 S6, M6, L6, and X6. Results concerning the performance figures in the previous points are shown in Figure 13.

As expected, reducing the model size from X to S leads to an increase of the TAF and the system experiences an improved real-time behavior. The TD value follows the TAF trend for S, M, and L configurations and it becomes practically steady for X models, both for 640p and 1280p. Considering only the 640p models, the highest value in TDA is reported by the YOLOv5 M version, and thus it would be preferable when the computational power is a limiting factor. In a real setup, the YOLOv5 S/M CNNs should be preferred due to achievable better performance in terms of the considered metrics (i.e., accuracy, recall, frame rate, etc.). In this case to guarantee real-time performance, GPU devices are required. In general, a trade-off between input resolution, computational resources and interesting performance metrics should be considered in the system design. Eventually, YOLOv5 S 640p, which has only 7.3 M parameters (compared to 21.4 M for the M version) represents still a good alternative for low-end systems.

Finally, to assess the required transmission capacity, in the Table 3 we report the bit rates of the videos streams to be sent to the remote PD [32,33]. Initially, both uncompressed and compressed cases were considered for variable video resolutions. We start considering a frame rate of 30 fps and assuming a variable video compression factor from 1:100 to 1:200 (Compression factors of the most popular codecs such as H.264, H.265, MP4 vary between 1:200 to 1:50). Results are shown in Table 3a. The uncoded case can be considered as viable only in the case of local processing of frames. From the analysis at the start of this Section, we observed that the considered PD based on YOLOv5 is not able to process all the frames from the webcam. This fact can be conveniently used to further reduce the video bit rates and then the required transmission capacity by decreasing the number of fps below 30 and adapting the compression factor in the cases corresponding to videos at high-resolution. Results were reported in Table 3b.

The achieved bit rate reduction can be significant also in case of 640×360 case by properly adapting the frame rate and the compression ratio, thus enabling the transfer of videos from the webcam to the remote PD over wireless connections.

## 7. ADAS Iatency and Braking Curve for the Vehicle

**Figure 14:**
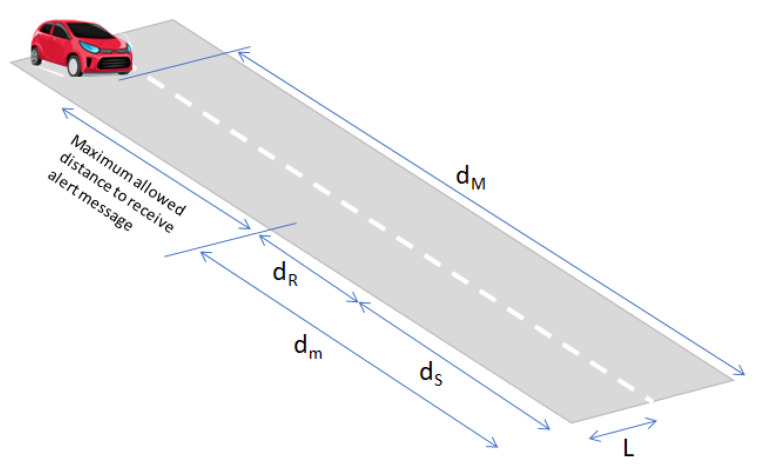
Simple geometrical scheme for calculation of maximum allowed distance from pedestrian to receive alert message to avoid accident with crossing pedestrian.

To provide an indication of the overall latency requirement on ADAS to prevent accidents of vehicles with pedestrians, we consider the geometrical scheme in Figure 14, showing a vehicle approaching the crossing point, while moving at constant speed *v* km/h. We assume the road is *L* m large and the pedestrian starts to cross it at speed $v_{p}$ m/s.

According to Figure 14, the maximum distance $d_{M}$ at which the car needs to be alerted of crossing is:

(11) $$d_{M}=v\cdot \frac{L}{v_{p}}$$

In principle, cars at distance $d>d_{M}$ from the crossing point and traveling at the same speed, the alert message could be unnecessary. The minimum distance $d_{m}$ from the crossing point to avoid collision with the pedestrian is:

(12) $$d_{m}=d_{R}+d_{S}$$

where $d_{R}$ is the distance traveled by the car during the time interval corresponding to the driver reaction time, i.e., $d_{R}=v\cdot \Delta t_{R}$ with $\Delta t_{R}$ is the reaction time of the driver. Typical values for $\Delta t_{R}$ vary from 0.5 s up to 1.5 s depending on the driver physical characteristics. The $d_{S}$ in (12) is the distance required for stopping the car. It can be estimated by the following approximated formula [34]:

(13) $$d_{S}=\frac{v^{2}}{2\beta }$$

where β is the deceleration during braking and β=6.9 m/s^2^ is the typical value in the case of emergency braking. As an example, for v=50 km/h we have $d_{S}≅14$ m, the time interval $\Delta T=(d_{M}-d_{m})/v$ s is the maximum time interval for the alert message to be sent by the assistance system to the car at distance $d_{M}$ in (11) from the pedestrian crossing point. Obviously, if the car is at distance *d* and $d_{m}<d<d_{M}$ from the crossing point, the available alerting time interval ΔT is reduced. For cars at distances lower than $d_{m}$ and traveling at speed *v*, in principle collision is inevitable. To have an idea for the values of ΔT, we consider one car at v=50 km/h, i.e., v≅14 m/s. From previous formula, assuming $v_{p}=1$ m/s and a reaction time of the car driver of 1 s (typical value) we obtain $d_{M}=14\cdot 3=42$ m and $d_{m}=14+14=28$ m so that $d_{M}-d_{m}=14$ m, corresponding to ΔT=14/14=1 s i.e., the alert message should be sent to the driver within ΔT. Previous formulas can be used to derive the braking curve required for regulating the speed of a vehicle approaching the crossroad area. In general, the system should guarantee that a car has enough time to stop after receiving a crossing alert from the camera.

Let $\tau_{T}$ be the overall latency required for generating and transmitting the alert message to the intended on-board terminal(s):

(14) $$\tau_{T}=\tau_{C}+\tau_{P}$$

where $\tau_{C}$ is given in accordance with the communication path in Section 5 and $\tau_{P}$ is the processing time that can be easily evaluated taking into account for the behavior of the proposed PD system detailed in the activity diagram in Figure 11. In particular, the $\tau_{P}$ is proportional to the inverse of the rate of processed frames which can be lower or equal to the video frame rate depending on the available computational power in the PD.

**Figure 15:**
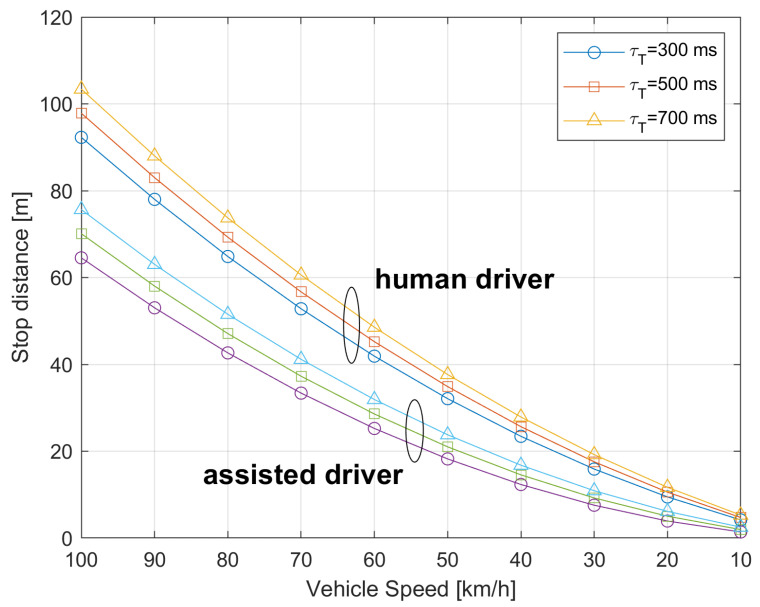
Distance for car stop as a function of the car speed for different overall latencies, $\tau_{T}$.

The distance *d* from the crossing point can be estimated by the app in the car on the basis of the position of the camera, the GPS and the speed information of the car. To guarantee the car to stop in the case of alert message issued by the camera, the car should regulate its speed so that:

(15) $$d\geq d_{R}+d_{S}+d_{\tau }$$

where $d_{\tau }=\tau_{T}\cdot v$ is the distance traveled by the car, while the alert message is still being transmitted to the app in the car. Equation (15) can be inverted with respect to *v* to obtain the speed required to stop at the crossing in the case of alert. From (15) in Figure 15, we plot the required distance for car stop as a function of the car speed *v*. Variable $\tau_{T}$ from 300 ms up to 700 ms was considered. Results refer to human driver with reaction time $t_{R}=1$ s and to an assisted driver with automated braking with $t_{R}=1$ ms.

As an example, assuming the car is at distance d=40 m from the crossing point, to stop the car (in the case of persistent alert) the human driver should be able to reduce its speed between 52 km/h (for $\tau_{T}=700$ ms) and 58 km/h (for $\tau_{T}=300$ ms). To avoid the car stopping when it is too close to the crossing point, we can identify a minimum distance $d_{min}$ from the crossing such that for $d\leq d_{min}$.

For the human driver case, we can assume that the car is proceeding at the corresponding constant speed and the driver is able to see the crossing point and act accordingly in the case of pedestrian crossing. In the case of automated assisted braking we could assume that for $d<d_{min}$ the verification of pedestrian crossing is left to car sensors, e.g., a car radar able to detect the presence of obstacles in the close proximity. As an example, from Figure 15 the $d_{min}$ could be set to $d_{min}=20$ m corresponding to a car speed lower than about 30 km/h for a human driver.

Final insights for the ADAS designer.

Before concluding, it is useful to give some insights to the designer for properly deploying the ADAS system. In the following, we propose a procedure for setting the ADAS parameters of the proposed system. In the first step, the designer has in input the specific crossroad characteristics. Then, based on the required metrics in (7)–(10) (e.g., a certain value of accuracy or recall), the designer should select the proper neural network for PD (e.g., processing capabilities, number of layers, etc.). The output of this step is the frame rate, which the PD is able to process (for example, 14 fps giving a processing time $\tau_{P}=71$ ms). In the third step, the designer has to input who is the manager of the ADAS system as well as the adopted communication system. The choices are among the local manager (i.e., architecture 3, thus considering the path C or path E), the network operator (i.e., architecture 1 or path A) or the external service provider, namely Over-The-Top (i.e., architecture 2, thus considering the path B, path D or path F). Based on the available communication system and properly selecting the latency threshold (e.g., at 90th percentile), the designer obtains the communication time $\tau_{C}$. In case of path B, $\tau_{C}=63.8$ ms. To reduce the false alarm in signaling a pedestrian crossing the road, it is possible to send the warning message only after at least three consecutive PD detections, i.e., after a pedestrian is detected three times in three consecutive analyzed frames. At this point, the overall latency $\tau_{T}$ is known (i.e., $\tau_{T}=280$ ms in the example). Based on the braking curve in Figure 15, the maximum vehicle speed can be transmitted to the app on the on-board device, considering the real position of the vehicle and its distance from the crossroad. This allows the driver or to the automated driving system to regulate the vehicle’s speed in accordance with the curve in Figure 15.

## 8. Conclusions

The problem of detecting crossing pedestrians to avoid collisions with incoming vehicles is an open and important issue for driver’s safety. In this paper, we proposed an Autonomous Driver Assistance System (ADAS) for fast warning drivers incoming in a crossroad area. The proposed system can provide the positions of pedestrians crossing or willing to cross the street to the intended drivers. The considered ADAS includes two subsystems: a convolutional neural network (CNN)-based Pedestrian Detector (PD), and a communication infrastructure used to forward alerts to drivers. We analyzed the characteristics of these two subsystems. In particular, we discussed three viable communication system architectures that can be used in ADAS, and we assessed the performance of the CNN-PD system based on YOLO CNN on a real hardware/software platform. Performance of the considered communication architecture for the ADAS were evaluated in terms of the communication time. A maximum transmission delay greater than one hundred of ms was estimated when public mobile radio networks are used for forwarding alert messages. These delays can be significantly reduced with the inclusion of Multi-Access Edge Computing (MEC) in the transmission/processing chain or by adopting the distributed communication architecture presented in this paper using local area radio technologies to forward alert messages.

Analysis on PD behavior shows that its accuracy varies from 70 to 100% depending on complexity of the videos and on the ability of the CNN to distinguish pedestrians from the rest of the image. We evaluated the performances of YOLOv5, concluding that the M size model can be a good trade-off between the accuracy and processing speed.

## Figures and Tables

**Table 1:** Minimum and maximum transmission latencies (ms) from the generation of the alert to its visualization in the on-board app.

	**Architecture 1**		**Architecture 2**		**Architecture 3**	
	**(Server EWS)**		**(Server BRK)**		**(Local Processing)**	
**LINKS**	**Path A**		**Path B**		**Path C**	
	*min*	*max*	*min*	*max*	*min*	*max*
APP processing	2	5	2	5	2	5
Cam-WiFi					1	2
WiFi-APP					10	50
Wired Access (cam-CO)	2	20	2	20		
Wired Access (CO-WiFi)						
Core Nwk (CO-EWS/	8	10	8	10		
CO-GW/CO-router)						
Core Nwk-Wireless Access	8	10	8	10		
(EWS-router/GW-router)						
Wireless Access (router-BBU)	2	5	2	5		
Air Interface DL (BBU-APP)	3	4.5	3	18		
Air Interface UL (cam-BBU)						
Internet (GW-BRK)			3	5		
Internet (BRK-GW)			3	5		
**Total [ms]**	**25**	**54.5**	**31**	**78**	**13**	**57**
	**Architecture 2**		**Architecture 3**		**Architecture 2**	
	**(MEC @BBU)**		**(MEC @CO,**		**(MEC @CO, Fixed/**	
			**Wi-Fi EDGE)**		**Mobile nw)**	
**LINKS**	**Path D**		**Path E**		**Path F**	
	*min*	*max*	*min*	*max*	*min*	*max*
APP processing	2	5	2	5	2	5
Cam-WiFi						
WiFi-APP			10	50		
Wired Access (cam-CO)			2	20	2	20
Wired Access (CO-WiFi)			2	20		
Core Nwk (CO-EWS/					8	10
CO-GW/CO-router)						
Core Nwk-Wireless Access						
(EWS-router/GW-router)						
Wireless Access (router-BBU)					2	5
Air Interface DL (BBU-APP)	3	18			3	18
Air Interface UL (cam-BBU)	3	18				
Internet (GW-BRK)						
Internet (BRK-GW)						
**Total [ms]**	**8**	**41**	**16**	**95**	**17**	**58**

**Table 2:** Results based on considered metrics.

**Clip Name**	**# Frames**	**# Samples**	**TP**	**FP**	**FN**	**TN**
*Simple scenarios*						
Left	1550	31	12	0	0	0
Zebra Crossing	1400	28	29	1	0	0
Right	3150	63	91	2	7	0
Multiple Directions	1850	37	84	0	5	0
*Real-life scenarios*						
Florida	2849	57	130	8	47	0
Av. Do Mar	2757	56	141	4	17	0
Rome	3406	69	361	3	11	0
**Clip Name**	**Precision**	**Recall**	**F-Measure**		**Accuracy**	
*Simple scenarios*						
Left	1.00	1.00	1.00		1.00	
Zebra Crossing	0.97	1.00	0.98		0.97	
Right	0.98	0.93	0.95		0.91	
Multiple Directions	1.00	0.94	0.97		0.94	
*Real-life scenarios*						
Florida	0.94	0.74	0.83		0.70	
Av. Do Mar	0.97	0.89	0.93		0.87	
Rome	0.99	0.97	0.98		0.96	

**Table 3:** Bit rate for uncompressed and compressed cases: variable fps values, 4 bytes per pixel (HD case).

**(a) fps = 30 [1/s]**				
		**Bit Rate,**	**Compression**	**Bit Rate,**
		**Uncoded [kbit/s]**	**Factor**	**Compressed [kbit/s]**
YOLOv5 640×640		393,216.00	200	1966.08
YOLOv5 640×360		221,184.00	150	1474.56
YOLOv5 340×200		65,280.00	100	652.80
**(b) Variable fps (According Yolo Processing Capabilities)**				
	**Variable**	**Bit Rate,**	**Compression**	**Bit Rate,**
	**fps**	**Uncoded [kbit/s]**	**Factor**	**Compressed [kbit/s]**
YOLOv5 640×640	27	353,894.40	200	1769.47
YOLOv5 640×360	27	199,065.60	200	995.33
YOLOv5 340×200	27	58,752.00	200	293.76
YOLOv5 640×640	14	183,500.80	150	1223.34
YOLOv5 640×360	14	103,219.20	150	688.13
YOLOv5 340×200	14	30,464.00	150	203.09
YOLOv5 640×640	7	91,750.40	100	917.50
YOLOv5 640×360	7	51,609.60	100	516.10
YOLOv5 340×200	7	15,232.00	100	152.32
